# Orbital inflammatory pseudotumor: new advances in diagnosis, pathogenesis, and treatment

**DOI:** 10.1186/s40001-023-01330-0

**Published:** 2023-10-04

**Authors:** Yenan Fang, Bingyan Shen, Qin Dai, Qiqi Xie, Wencan Wu, Min Wang

**Affiliations:** 1 National Clinical Research Center for Ocular Diseases, Eye Hospital, Wenzhou Medical University, Wenzhou, Zhejiang People’s Republic of China; 2 State Key Laboratory of Ophthalmology, Optometry and Vision Science, Eye Hospital, Wenzhou Medical University, Wenzhou, Zhejiang People’s Republic of China

**Keywords:** Orbital inflammatory pseudotumor, Diagnosis, Etiology, Medical treatment, T cells

## Abstract

Orbital inflammatory pseudotumor (OIP) is a benign, non-specific inflammatory disorder that commonly occurs in middle-aged adults and is usually unilateral but can occur bilaterally. Its clinical manifestations have tremendous clinical heterogeneity and vary according to the site of infiltration and the degree of lesions, including orbital pain, swelling, diplopia, proptosis, restricted eye movement, and decreased visual acuity. Clinical features, Image characteristics and pathological examinations often need to be evaluated to confirm the diagnosis. Currently, there is no systematic research on the pathogenesis of OIP, which may be related to immunity or infection. The first-line treatment is glucocorticoids. Radiotherapy, immunosuppressants, and biologics can be considered for treatment-resistant, hormone-dependent, or intolerant patients. In this review, we aim to summarize and focus on new insights into OIP, including new diagnostic criteria, pathogenesis, and discoveries in new drugs and treatment strategies. In particular, we highlight the literature and find that T cell-mediated immune responses are closely related to the pathogenesis of OIP. Further exploration of the mechanism and signaling pathway of T cells in the immune process will help to identify their therapeutic targets and carry out targeted therapy to treat refractory OIP and reduce the side effects of traditional treatments.

## Introduction

Orbital inflammatory pseudotumor (OIP), also known as Idiopathic orbital inflammation (IOI) and idiopathic orbital inflammatory syndrome (IOIS), is a benign, noninfectious, non-specific orbital inflammation [1, 2]. It has the third highest incidence among adult orbital diseases, after Graves’ ophthalmopathy and lymphatic proliferative diseases [2, 3].

The disease has been reported in all ethnic groups worldwide. Its incidence is inconsistent in different reports, and the following are some extensive sample research data. Of the 1264 patients referred to the ocular oncology department for space-occupying orbital lesions at the Wills Eye Hospital in Philadelphia, USA, 133 (11%) were ultimately pathologically confirmed to have inflammatory lesions [4]. A retrospective study of 6328 patients with the orbital disease treated at Aravind Eye Hospital between January 1997 and December 2008 showed that 1473 (23%) had OIP [5]. Among 1000 patients with primary orbital tumors clinically or histopathologically diagnosed at Tokyo Medical University Hospital from 1995 to 2019, OIP accounted for 27% and was the most common benign orbital tumor [6]. OIP can involve the lacrimal gland, sclera, extraocular muscle, optic nerve sheath, etc., mostly confined to the orbit. Still, it also extends to adjacent structures outside the orbit, such as the sinus, in some cases, and occasionally invades intracranial structures [7–9].

The diagnosis of OIP can be challenging in clinical practice as presentation is highly variable. Most of the traditional diagnostic methods were based on the combination of clinical manifestations, such as orbital pain, eyelid swelling and ocular mass, imaging examinations and pathological biopsy experience, and there was little consensuses on the diagnosis [10]. It is currently believed that OIP presents as a nonspecific pleomorphic invasion, which is confirmed by histopathology. Well-differentiated lymphocytes, neutrophils, macrophages and eosinophils can be seen in the tissue [11].

The exact etiology of OIP is still inconclusive. Previous experimental results have shown that abnormal immune response activation exists in OIP. Still, some researchers have also found that it is related to infection with pathogens, such as Epstein–Barr virus [12, 13]. With further research, the role of abnormal T cells in the pathogenesis of OIP has gradually attracted attention, which may be the cause of an abnormal immune response and immune imbalance [14]. However, the specific impact mechanism of T cells in OIP needs to be summarized and analyzed. Although natural remission occasionally occurs without treatment, glucocorticoids have been the cornerstone of therapy in the acute phase. Responses to treatment are generally favorable, but relapse, hormone dependence, and side effects cause enormous emotional stress and a great burden on patients' mental health and quality of life [15]. Immunosuppressants, radiation therapy, and novel biological therapies offer additional options for treating the disease, each with pros and cons. This review is an update of the understanding of OIP diseases, including the diagnostic consensus, pathogenesis and treatment methods. It is worth noting that thanks to the development of molecular immunology and sequencing technology, we found the pathogenesis of OIP is likely to be related to T cell-mediated immunity, which may be the direction of future research.

## Etiopathogenesis

The pathogenesis of OIP has not been thoroughly studied, and its underlying pathophysiological mechanisms remain largely unknown. In recent years, with the progress of scientific research, more and more studies have tried to explore or hypothesize the pathogenesis of OIP. The two main viewpoints are autoimmune theory and viral infection theory. Immune-mediated pathophysiological mechanisms have been more widely recognized, but no complete and detailed hypothesis has been proposed.

### Autoimmunity theory

Autoantibodies reactive with ocular muscle antigens were detected in the serum of most OIP patients, suggesting that OIP probably had autoimmune responses against specific retro-orbital antigens [16]. Wladis, et al. used immunohistochemical staining and found that toll-like receptors (TLRs) are present in OIP biopsy specimens, while controls did not demonstrate any TLRs [17]. Toll-like receptors are a family of membrane-spanning proteins. These receptors recognize pathogen-associated molecular patterns, and trigger innate immune responses or initial antigen-specific adaptive immunity [18, 19]. Therefore, it can be inferred that OIP is closely related to an abnormal immune response.

Circulating fibroblasts have characteristics of immune cells and fibroblasts. They are derived from bone marrow-derived stem cells and then recruited from the circulating blood to sites of injury and inflammation [20, 21]. Circulating fibroblasts are involved in the fibrotic process of diseases, such as asthma, idiopathic pulmonary fibrosis and chronic renal fibrosis, and are significantly elevated in certain autoimmune diseases, such as Graves' ophthalmopathy and rheumatoid arthritis [22–26]. Recent studies have found that the expression of CD40 on the surface of fibroblasts of patients with OIP was considerably higher than that of normal donors, and fibroblasts of all patients expressed IL-6 when stimulated [27]. It is inferred that fibroblasts may play a role in orbital inflammation and fibrosis in OIP. Furthermore, CD40-mediated production of activating cytokines may contribute to the pro-inflammatory and pro-fibrinogenesis of OIPs. Fibrosis is an essential component of the inflammatory response and is involved in the process of orbital inflammation, and is thought to impact prognosis negatively [28]. Through pathological analysis, the researchers found that fibrosis in orbital adipose tissue was significantly increased in OIP subjects. Furthermore, fibrosis-related transcripts such as fibronectin, thrombospondin, lumican, and collagen types I and VIII were found to be upregulated in OIP by the gene microarray approach [29]. The above research results provide a reference for the search of anti-fibrosis drug targets, and also provide a direction for targeted therapy of the disease.

Abnormalities in the molecular biologic milieu have been reported in OIP. A research team from Albany Medical College found six cytokines that were significantly increased in OIP biopsy specimens (IL-2, IL-8, IL-10, IL-12, gamma interferon, and tumor necrosis factor alpha). Specifically, IL-12 promotes the production of T helper 1 (Th1) cells involved in cell-mediated inflammation and delayed-type hypersensitivity, while IFN-γ is the main product of Th1 cells [30, 31]. This inferred a significant pathogenetic role of Th1 in the pathogenesis of OIP. Rui et al. used human oligonucleotide microarrays to analyze the gene expression profiles of OIP and normal tissues and found some interesting differences in gene expression [32]. First, many cytokines and cytokine receptors associated with inflammation were highly expressed in OIP, suggesting that OIP is a nonspecific inflammatory response. In addition, T and B lymphocyte activation and proliferation-related factors were significantly upregulated in OIP. Factors mainly expressed on the surface of antigen-presenting cells and activated T cells, such as HLA-DRB1 and HLA-DQA1, were also found to be dramatically increased in OIP. This implied that OIP is closely related to the immune response, especially the T cell-mediated immune response.

Evidence of other autoimmune regulatory imbalances has also been found in OIPs. Chen et al. found that regulatory T cells (Tregs), which were considered as a subgroup of suppressor cells involved in balancing immune responses by suppressing excessive immune responses, were increased in the circulating blood and afflicted orbital tissues of OIP patients compared with healthy individuals [14, 33, 34]. Notably, Tregs with proinflammatory and profibrotic polarization were increased in the OIP, presumably playing a role in this disease. Interestingly, interleukin 33 (IL-33) inhibits the pro-fibrotic and pro-inflammatory effects of Tregs, providing a potential therapeutic target for OIP. Dendritic cells (DCs) are the quintessential antigen-presenting cells (APCs) of the immune system and can be divided into plasmacytoid dendritic cells (pDCs) and conventional dendritic cells (cDCs) [35]. DCs can initiate specific T cell responses and are also able to tolerate T cells, thereby preventing self-reaction. In cancer therapy, DCs have been used as cellular vaccines designed to initiate patient antitumor responses [36]. The cDCs have been recognized as essential for identifying intracellular and extracellular pathogens and delivering exogenous antigens to T cells [37]. As to pDCs, they have been considered to regulate B cell differentiation and immunoglobulin secretion via CD70 and IL-6 [38]. Laban et al. used flow cytometry to find that the percentages of (HLA-DR+CD303+CD123+) pDCs and (HLA-DR+CD11c+CD1c+) cDCs type-2 in the peripheral blood of OIP patients were significantly lower than those in healthy individuals. Similarly, transcriptome analysis of orbital biopsy showed a reduced abundance of pDC and cDC populations in the OIP microenvironment [39]. The specific mechanism of the reduction in dendritic cells in OIP is unclear and may be related to etiology. Furthermore, its decline in cancer may be explained as immunosuppression caused by metabolic stress and hypoxia in the tumor microenvironment, which may be a reference for its role in OIP [40].

Research on the pathogenesis of OIP is diverse and broadly focused on abnormal changes in cells involved in the immune response, as well as increased pro-inflammatory factors and fibrosis. Abnormal immune responses are manifested in the activation of T cells and B cells, an increase in regulatory T cells, and a decrease in dendritic cells. These changes disrupt the balance of the immune system and lead to abnormal immune responses. Although the detailed relationship between inflammatory immune response and the fibrotic outcome has not been uniformly determined according to the current findings, we have summarized some commonalities from the above studies. It is worth noting that most of the above reports point to the pathogenesis of OIP being closely related to the mediation of T cells. T cells are critical effector cells of the adaptive immune system, recognizing antigens through the membrane protein T cell receptor (TCR). Many TCRs in the human adaptive immune system are collectively referred to as the TCR repertoire. With the development of modern sequencing technology, the research on the T cell receptor repertoire has made rapid progress [41, 42]. Several bioinformatic approaches have addressed the diversity of T cell receptors. In autoimmune diseases, such as psoriasis vulgaris, systemic lupus erythematosus, and severe acne, TCR sequencing and bioinformatics analysis found that the diversity of the CDR3 sequence was different from that of the normal control group [43–45]. There is currently no report of TCR sequencing for OIP, which may be a direction for future exploration of the link between their immune lineage and disease. Through this method, it is expected to find further the T-cell clonotypes related to OIP, and further elaborate the specific process of the immune response.

### Viral infection theory

In addition to the classical immune theory, some scholars believed that OIP might be secondary to or associated with viral, bacterial or other pathogen infections, such as Epstein–Barr virus (EBV), Streptococcus, HIV, herpes zoster, and Leishmania braziliensis, among which EBV is mainly reported [46–50].

EBV infection can cause severe lymphocytic infiltration and has been shown to be associated with OIP. Spatially, positive signals for EBV-encoded small RNAs (EBERs) were found in the nuclei of activated immunoblasts and in small and medium lymphocytes between or around follicles [12]. However, it is thought-provoking that a study found that all OIP plasma samples were EB-VCA-IgG positive (16/16), while the healthy controls also had a positive rate of 100% (20/20). No EB-IgM positivity was detected in either OIP patients or healthy subjects [51]. There are also results contrary to the above findings, Leo suggested that EBV infection did not seem to be associated with OIP in Caucasians, because none of the OIP blood samples (0/4) could find EBV-DNA [52].

There are few studies on the specific mechanism of viral role in OIP, but hypotheses have been proposed in other autoimmune diseases, such as Graves’ ophthalmopathy. Keiko Nagata attempts to explain the immune response to Epstein–Barr virus infection in Graves’ ophthalmopathy. This disease is a thyroid associated disease with characteristic autoantibodies against thyroid components, TRAb. It is speculated that the reactivation of persistent EBV in TRAb-positive B cells induces the production of IgM autoantibodies and activates the classical complement pathway to produce TRAb to induce or aggravate Graves’ ophthalmopathy [53].

In conclusion, traces of EBV infection in the circulating blood or in involved tissues can be detected in the vast majority of OIP patients. However, EBV infection status is not specific, and there is no evidence that OIP is directly related to recent EBV infection. The mechanism of action of EBV in OIP has not yet been reported, and its mechanism speculation in Graves' ophthalmopathy can be used as a reference.

### Others

Although the nature of the non-specific inflammatory response to OIP has been recognized, its pathophysiological manifestations remain to be explored. In recent years, with molecular biology, some scholars have proposed new possible pathogenic mechanisms. MicroRNAs (miRNAs) are small noncoding regulatory RNAs whose compositional changes are associated with human pathophysiological processes, such as inflammation and cancer, etc. Bashant et al. identified a paninflammatory miRNA-cluster, especially miR-223, that was upregulated in patients with OIP compared to controls [54]. MiR-223 derived from neutrophils in blood is a significant source of serum miRNAs [55]. In addition, a positive correlation was shown between the size of neutrophil cells and serum miR-223 levels in OIP patients [54]. These analyses suggested that changes in blood leukocyte composition may underlie increased miRNA clusters in blood in patients with OIP and that serum miRNA clusters correlate with neutrophil status. Furthermore, genes related to the PI3K–AKT pathway and the NF-κB pathway were found to be activated in OIP samples, suggesting that these two signaling pathways may play an essential role in its pathogenesis [56]. Interestingly, glucocorticoids can inhibit the NF-κB pathway by inducing the expression of IκB to increase the cytoplasmic retention of NF-κB, inhibiting the DNA binding activity of NF-κB, or competing with NF-κB for binding [57, 58]. This implied that the NF-κB pathway might be the target of the action of glucocorticoids in the treatment of OIPs.

## Diagnosis

Clinically, the diagnosis of OIP is usually based on a detailed medical history, clinical manifestations, ultrasonography, magnetic resonance imaging (MRI), computed tomography (CT) imaging findings and pathologic examination. To some extent, OIP is a diagnosis of exclusion. Ilse Mombaerts et al. used the modified Delphi approach to reach a consensus on the diagnostic criteria, making the diagnosis relatively uniform. According to the consensus, clinical indicators, MRI, or CT studies, selected normal laboratory findings, and incisional biopsy are instructive in diagnosing OIP [10].

### Clinical manifestations

OIP can be acutely, subacutely, or chronically usually in a unilateral orbit, but bilateral diseases can also occur simultaneously or sequentially [1, 59, 60]. The average age of patients is 39–45 years, but OIP can affect people of almost any age [1, 61]. OIP is slightly more prevalent in middle-aged females [62]. The presenting symptoms and signs of OIP may include orbital pain, swelling/mass, erythema, diplopia, proptosis, limited ocular motility, decreased vision, optic neuropathy, conjunctival congestion and ptosis [1, 63]. OIP can be divided into the following categories according to different anatomical sites. The typical clinical and morphological characteristics of OIP according to different anatomical locations are as follows (Table 1).

**Table 1:** Different categories of OIP [1, 10, 11, 64–66]

Clinical classification	Typical clinical features	Imaging characters	Pathological features
Lacrimal gland	Local pain, tenderness, eyelid swelling	Enlargement of the lacrimal gland, periglandular tissue inflammatory reaction	Infiltration of chronic inflammatory cells, fibrous connective tissue hyperplasia
Sclera and adjacent tissue	Orbital pain, swollen eyelid, inflammation	Enlargement of the scleral uveal rim, blurring of the sclera margin and adjacent tissues	–
Extraocular muscles	Painful and restricted eye movement, diplopia	Diffuse enlargement of the extraocular muscles with blurred edges, especially the medial rectus	Muscle fiber expansion, mild endomysial fibrosis
Optic nerve sheath	Loss of visual, decreased color vision associate with pain	Enlarged optic nerve sheath, indistinct borders, and surrounding fatty infiltration	–

### Imaging features

Different categories of OIP have various imaging manifestations. When the lacrimal gland is involved, CT shows that the density of OIP is similar to or slightly greater than that of muscle and can be significantly enhanced. The OIP on MRI shows a slightly lower or equal signal than the brain, with uniform enhancement on the enhancement sequence. OIP of the sclera and adjacent tissues showed nonspecific thickening structures on imaging with a blurring tissue margins. When the extraocular muscles are involved, imaging is similar to myositis, with enlargement of the tendons and muscles of the extraocular muscles, as well as thickening, blurring, and enhancement of their attachment sites. The radiological manifestations of OIP include mass compression around the optic nerve sheath and blurring of the optic nerve sheath edge, and contrast-enhanced CT and contrast-enhanced MRI images show enhancement of the optic nerve sheath in contrast to the central hypodense nerve. Moreover, as one of the most commonly used sequences in brain MRI, diffusion-weighted imaging (DWI) has been increasingly used to characterize the differential diagnosis of solid orbital masses and provides quantitative information in the form of the apparent diffusion coefficient (ADC). Distinguishing between lymphoma and OIP is a common diagnostic challenge, and DWI has proven helpful for the vast majority of lymphomas and inflammatory lesions with no overlap in ADC values [1, 67]. Recently, Kamil G. Laban. et al. described zirconium-89-labelled rituximab PET–CT in aiding the diagnosis of orbital inflammation. A total of 5/8 patients had a strong 89 Zr-rituximab uptake [68].

### Pathological features

In terms of pathology, OIP is mainly characterized by infiltration of a variety of chronic inflammatory cells, including lymphocytes, plasma cells, and eosinophils, accompanied by varying degrees of fibrous connective tissue hyperplasia, without granulomatous inflammation, vasculitis, or necrotic areas. Plasma cell IgG4-positivity of ≤ 30 cells per high-power field (HPF), or IgG4 + /IgG ratio ≤ 40% is considered compatible with a diagnosis of OIP [10]. In addition, idiopathic orbital myositis, as a particular type of OIP, showed muscle fiber expansion due to inflammatory infiltration, mild endomysial fibrosis, and no granuloma or vasculitis as seen in any specimens [66, 69].

Besides, whether to perform an orbital biopsy is a matter of debate [13, 70]. The choice should be made according to the actual situation. When the clinical manifestations are relatively typical and the drugs are effective, there is no need for a biopsy, just drug therapy. When clinical manifestations and imaging findings are inconclusive, and the condition does not improve or worsens with conventional treatment, tissue biopsy can be performed using minimally invasive methods to exclude other diseases, such as lymphoma. Biopsies are usually performed for palpable orbital lesions, such as those involving the lacrimal gland. Pathological findings should be interpreted with clinical, imaging, and serological findings [13, 70, 71]. For myositis and the lumps located deep in orbit, a biopsy may damage the optic nerve. Therefore, a reasonable choice should be made regarding whether to perform a biopsy [2, 72–74].

### Laboratory examination

Laboratory tests for OIP generally include routine blood tests, electrolytes, erythrocyte sedimentation rate, C-reactive protein, sedimentation rate, antinuclear antibodies, antineutrophil cytoplasmic antibodies, and rheumatoid factor tests [75, 76]. When only the lacrimal gland is involved, additional blood sampling for the detection of anti-citrullinated protein antibodies and anti-cyclic citrullinated peptide antibodies is feasible. When extraocular muscles are involved alone, testing for thyroid hormone, triiodothyronine, thyrotropin, and thyroid-stimulating hormone receptor antibodies is recommended [10]. However, inflammatory markers and autoantibodies are sometimes negative and non-specific in OIP, so laboratory tests often do not have a clear disease direction. Overall, OIP is a diagnosis of exclusion.

### Differential diagnosis

#### Orbital lymphoma

Orbital lymphoma is a common orbital malignancy in adults, the majority of which are of B-cell origin [77]. When imaging is challenging to distinguish OIP from lymphoma, immunological detection and analysis can be used to distinguish the polyclonal proliferation of OIP from the monoclonal proliferation of lymphoma [78].

#### Graves’ ophthalmopathy

Graves’ ophthalmopathy is an autoimmune disease that is overwhelmingly associated with abnormal thyroid function [79]. OIP involves hypertrophy of the entire extraocular muscle, including the tendon and the muscle belly, whereas in Graves' ophthalmopathy the extraocular muscle involvement is limited to the muscle belly and there is diffuse fatty hyperplasia of the orbit [75].

#### Orbital cellulitis

Orbital cellulitis mostly originates in the paranasal sinuses and may be accompanied by systemic symptoms, elevated peripheral blood leukocyte counts, and sensitivity to antibiotic therapy [1, 75]. It can be distinguished from OIP by medical history, clinical manifestations and imaging examination.

### OIP in children

OIPs are relatively uncommon in children and need to be differentiated from rhabdomyosarcoma, lymphangioma and dermoid tumor [80, 81]. The five most common symptoms in pediatric OIP patients are palpable mass, limited eye movement, eyelid swelling, exophthalmos, and high intraocular pressure, sometimes accompanied by ptosis [82]. In children, OIP sometimes precedes the onset of certain systemic inflammatory diseases, including Crohn’s disease, Wegener’s granulomatosis, Churg–Strauss syndrome, and pauciarticular juvenile idiopathic arthritis [83–85].

### Others

In recent years, some studies have aimed to provide diagnostic information for OIP by analyzing the gene expression profile in biopsy tissue, combined with pathological detection to improve the diagnostic accuracy of OIP. Rosenbaum et al. compared five pathologically proven orbital adipose tissue, OIP, granuloma with polyangiitis (GPA), Graves’ ophthalmopathy, sarcoidosis and controls using RNA extraction and microarray analysis, principal coordinates analysis (PCA) to reveal differences in gene expression profiles between disease groups, and a Gene Set Enrichment Analysis (GSEA) program to identify genes that are highly expressed in microarray clusters [86]. Compared with the other four types, more heterogeneity was shown in the OIP sample, suggesting that OIP is not a single disease entity [87]. A comparison of OIP and GPA gene expression found no statistically significant differences [88]. Interestingly, when the test samples were replaced with lacrimal gland tissue for detection and comparison using the above method, similar conclusions were drawn; namely, there was significant heterogeneity in gene expression profiles in OIP, and 32% of OIP samples could not be distinguished from healthy control samples [89]. From this point of view, OIP has a variety of manifestations in gene expression, and there are some similarities with other diseases. Therefore, it is still necessary to combine clinical history, clinical manifestations, imaging examinations and pathological findings to diagnose this disease.

## Treatment

Traditional therapies such as glucocorticoids, radiation therapy, and immunosuppressive agents are effective in treating most OIPs and are still the mainstream. However, there are many cases of recurrence, posing challenges to treatment [76, 90]. In recent years, scientists have found that new drugs, such as biological agents, have good efficacy in the treatment of OIP, which brings more options for the treatment of refractory and relapsed OIP.

### Glucocorticoids

Currently, the recognized first-line treatment is glucocorticoids, and no specific targeted drugs have been developed. The advantages of glucocorticoids are that they are inexpensive, readily available, and relatively effective in treating OIPs. When they are effective, glucocorticoids typically have a rapid onset of action, improving signs and symptoms within a few days, which distinguishes hormone-sensitive patients from unresponsive or atypically responsive patients. Combination immunosuppressants or biological agents should be considered in patients who do not respond to glucocorticoids. However, glucocorticoids have relatively large side effects, and long-term use may lead to osteoporosis, peptic ulcer, diabetes, high blood pressure, electrolyte imbalance, etc. Regular medication can reduce the risk of side effects [70, 91, 92]. OIP tends to recur after glucocorticoid withdrawal, especially in patients with multiple muscle involvement. Rapid recurrence of symptoms after discontinuation of glucocorticoids may suggest multiple recurrences [59].

Systemic corticosteroid therapy can be administered orally or by intravenous infusion. In the Massachusetts Eye and Ear Infirmary, Boston, high-dose oral glucocorticoids (1.0–1.5 mg/kg/day) are usually administered for 1–2 weeks in the treatment of OIP, tapering over the next 5–8 weeks. Of 65 OIP patients treated with glucocorticoids, 63% had complete symptom relief, and 37% had partial or no symptom relief. Incomplete remission was due to the relapse of inflammation after a period of quiescence (58%) and relapse of persistent, refractory inflammation (38%) [62]. In the Eye Hospital of the Zhongshan Ophthalmic Center at Sun Yat-sen University, Guangzhou, China, systemic dexamethasone of 10 to 15 mg (0.2 mg/kg per day) is usually given intravenously for 3–5 days in the acute phase of OIP. After that, oral prednisone 0.8–1.0 mg/kg per day was administered for 2 weeks, and then the dose was reduced by 5 to 10 mg every 2 weeks. When the dose was reduced to 5 mg, it was maintained for 2 weeks and then withdrawn. Among 44 patients who received systemic glucocorticoid (prednisone or dexamethasone) therapy for orbital myositis, 38.6% recovered completely, and 59.1% partially recovered, with an average of 6.4 recurrences. The recurrence rate was 81.8% [61].

Some studies have shown that intraorbital or periorbital injection of glucocorticoids (such as triamcinolone acetonide) is an effective method for treating OIP and can reduce the systemic adverse reactions, which may be considered as first-line therapy in some patients. With regard to dose, 20 to 40 mg/mL of triamcinolone acetonide is most commonly used clinically. The injection can be repeated at 4-week intervals if the resolution has not been achieved [93–95].

In conclusion, glucocorticoid therapy has the advantages of fast onset and noticeable symptom relief, but its recurrence rate is high, and long-term use has many potential side effects.

### Radiation therapy

Radiation therapy has usually been used in patients with OIP who are refractory, hormone-dependent, or intolerant to systemic glucocorticoid therapy. Commonly used radiation treatments include en face electron, intensity modulated radiation, and opposed lateral field three-dimensional conformal radiation [73].

Bruno Fionda, et al. analyzed the literature on OIP radiation therapy published between 1978 and 2018 and found that the initial treatment response rate was 74–100%, with positive effects of treatment appearing within 1–2 months. The median recurrence rate was 10%. The median total dose was 20 Gy, which is the same as the most commonly used low-dose radiation therapy dose, often 2 Gy per day over 10 days. Side effects are less common in reports, mainly including cataracts, increased or decreased tear secretion, and photophobia [61, 75, 96, 97]. Older patients with a complete response to radiation therapy had a lower recurrence rate of OIP [73]. The short-term effective rate of OIP radiotherapy is high and relatively safe, but the long-term control is not very satisfactory.

### Immunosuppressants

Immunosuppressive therapy can be used in patients who are insensitive to glucocorticoid therapy. Commonly used drugs are cyclosporine-A (CsA), methotrexate, cyclophosphamide, azathioprine, mycophenolate mofetil, mycophenolate mofetil, and so on [98, 99].

#### Cyclosporine-A (CsA)

Gumus et al. reported that CsA had a good effect on refractory OIP. CsA inhibits lymphocyte-mediated responses and inhibits T cells activation by inhibiting the dephosphorylation of nuclear factors of activated T cell, so that they cannot enter the nucleus, thereby interfering with interleukin-2 production [75, 100]. These mechanisms support the idea that T lymphocyte-mediated immune responses play an essential role in the pathogenesis of OIP. Both oral cyclosporine 4 mg/kg per day for 6 weeks and topical CsA 0.05% eyedrops (Restasis) have been reported to be effective for OIP. However, side effects such as renal dysfunction, high blood pressure, gingival hyperplasia, and increased hair growth may occur when cyclosporine A is used. Therefore, the patient's kidney function must be closely monitored [75, 100].

#### Methotrexate

Smith et al. reported that the use of low-dose methotrexate in treating OIP had relatively few adverse reactions and was well-tolerated [101]. Methotrexate inhibits the synthesis and metabolism of purine and purine nucleotides by inhibiting dihydrofolate reductase and acts on the DNA synthesis phase to inhibit rapid cell division, thereby inhibiting the function of B cells and T cells [75, 100]. It has been reported that the initial dose of methotrexate at 7.5 mg/week and gradually increasing to 15 mg/week to 25 mg/week under the condition of tolerable side effects can bring clinical benefit to most patients. When using methotrexate, it is necessary to monitor liver enzyme levels, limit alcohol consumption, and supplement with adjuvant folic acid to reduce adverse effects, such as fatigue, gastrointestinal discomfort, hair loss, arthralgia, headache and abnormal liver function [75, 102].

#### Tacrolimus

Tacrolimus inhibits T cell activation and T cell-dependent B cell activation by forming a complex with immunophilin-FK-binding protein to inhibit calcineurin. It has been reported to be used in organ transplantation, ulcerative colon inflammation, rheumatoid arthritis, polycystic ovary syndrome, and myasthenia gravis. The might side effects are hypertension, neurotoxicity, diabetes, and nephrotoxicity [103–106]. Hyun Jae Kim et al. reported that tacrolimus may be a secure and efficient immunosuppressant for the treatment of refractory or relapsed OIP [107].

In conclusion, immunosuppressants can be used as an effective second-line drug for treating OIP. When using it, attention should be paid to its side effects, and blood biochemical indicators should be checked regularly to minimize systemic damage.

### Biologic agents

With the vigorous development of biomedicine, an increasing number of new options, such as infliximab, rituximab, adalimumab, etanercept, daclizumab, abatacept, and tocilizumab, are available for patients with refractory OIP. Potential efficacy, high cost of biologics, possible adverse effects of treatment, and even unanticipated complications should be fully balanced before patients with refractory OIP are treated with biologics [108]. The biologic agents clinically used in OIP mainly include infliximab, rituximab, tocilizumab, adalimumab and etanercept [75, 109–114].

#### Infliximab

Infliximab is a chimeric (murine-human) monoclonal IgG1κ antibody that binds both circulating and membrane-bound TNF-α. It is a hybrid antibody produced from human and murine amino acids that binds to tumor necrosis factor-a. It inhibits the differentiation of Th1 and Th2 cells by inhibiting the activation of the downstream nuclear factor-kB signaling pathway by preventing the production of pro-inflammatory cytokines by tumor necrosis factor-α [99, 115–117]. Infliximab has been reported for the treatment of autoimmune diseases, such as ankylosing spondylitis [118, 119], Crohn’s disease [120], ulcerative colitis [121, 122], Behcet’s disease [123], rheumatoid arthritis [118] and sarcoidosis [124]. The usual dose of infliximab is 3–5 mg/kg at 0, 2 and 6 weeks and every 4–8 weeks thereafter, which costs $12,000 to $18,000 per year [125]. Common side effects of infliximab include rash, headache, and low blood pressure. Serious but extremely low incidence side effects include the potential risk of tuberculosis and invasive aspergillosis, worsening of moderate-to-severe chronic heart failure, and demyelinating disease [126–129]. Overall, infliximab was generally well-tolerated and has been shown to be effective at treating OIPs. However, given its higher cost, we tend to use it only in refractory patients.

#### Rituximab

Rituximab is a human/mouse chimeric monoclonal antibody synthesized by genetic engineering. One mechanism mediating B-cell depletion is its binding to CD20 on B lymphocytes, leading to B-cell lysis and clearance through antibody-dependent and complement-dependent cytotoxicity. Several systemic autoimmune and orbital inflammatory diseases, such as autoimmune hemolytic anemia, Behcet’s disease, Graves’ ophthalmopathy and granulomatosis with polyangiitis, have been found to be effective with rituximab [130–132]. Rituximab has been shown to be effective in refractory OIP, such as those that are resistant to glucocorticoids, refractory to surgery or radiation therapy [110, 133–135]. It is relatively safe, and adverse reactions mainly include allergic reactions, cytokine release syndrome, infusion reactions, gastrointestinal symptoms and serum sickness, etc. Severe adverse reactions such as infections during B cell depletion are rare [108, 131].

#### Tocilizumab

Tocilizumab, also known as myeloma receptor antibody, is a recombinant humanized monoclonal antibody against human immunoglobulin G1. It targets the soluble and membrane-bound interleukin-6 receptor, a multifunctional pro-inflammatory cytokine implicated in a variety of inflammatory and autoimmune diseases [136]. Tocilizumab is licensed in the EU for rheumatoid arthritis, systemic juvenile idiopathic arthritis, polyarticular juvenile idiopathic arthritis and polyarticular juvenile idiopathic arthritis. In recent years, there have been case reports of long-term stable remission with tocilizumab in the treatment of refractory OIP [111, 137]. It was well-tolerated at long-term follow-up, and common complications of treatment included elevated cholesterol levels, gastrointestinal symptoms and mildly elevated liver enzyme levels [138, 139].

#### Adalimumab

Adalimumab is a TNF-α inhibitor that is essentially a recombinant human immunoglobulin G1 monoclonal antibody. It modulates downstream processes mobilized or regulated by TNF-α by blocking its interaction with p75 and p55 cell surface TNF receptors after binding to TNF-α [140]. Adalimumab has been used in autoimmune diseases, such as psoriasis, Crohn's disease, and noninfectious uveitis [141–143]. Although not widely used, adalimumab has been reported in adults and children to treat OIP, which can relieve symptoms and reduce the dose of glucocorticoids. Common side effects are redness and itching at the subcutaneous injection site. Infrequent reactions such as infections, nasopharyngitis, arthralgia, lupus-like syndromes, central nervous system demyelinating disorders, and progression of lymphoma have also been reported [99, 114, 144, 145].

As a product of the development of medical pharmacy in the new era, a large number of studies have reported that biological inhibitors are effective and relatively safe for refractory OIP. However, the prediction of its application effectiveness is still lacking, and the selection should be made after fully considering the patient’s economic level, psychological expectations and the ability to accept serious adverse reactions.

### Others

Plasma exchange therapy has been recognized to achieve satisfactory results in treating some immune-mediated neurological diseases that are ineffective in drug therapy, such as chronic inflammatory demyelinating polyneuropathy, Guillain–Barré syndrome and monoclonal gammopathy of undetermined significance [146]. OIP is known to be associated with humoral-cell-mediated immune complex processes [147]. A case of refractory OIP resistant to hormones and immunosuppression was recently reported, in which clinical symptoms were significantly relieved after plasmapheresis. It is speculated that the elimination of antibodies and other humoral factors by plasma exchange may reduce OIP recurrence [148].

Curcumin has the ability to regulate various signaling molecules, such as pro-inflammatory cytokines, NF-κB, C-reactive protein and ET-1. It has been used as a safe and effective drug for the treatment of OIP [149, 150].

Surgical treatment is suitable for well-defined and fibrosclerotic OIP masses that do not respond well to conservative treatment [28, 151, 152]. For patients with relatively limited masses and clear borders on imaging, the lesions can be removed at one time, and pathological examinations can be performed simultaneously [28, 151, 152].

## Conclusion

OIP is a common non-specific inflammation in ophthalmology, and its mechanism has not been thoroughly studied. It is crucial to update the information about its diagnosis, treatment and pathogenesis in time. Diagnosis should be made after comprehensive consideration of detailed medical history, clinical manifestations, imaging, pathological results, and laboratory results. Exploration of its pathogenesis, is currently more inclined to the immune theory, rather than viral infection without direct evidence. Studies have shown that it may be closely related to T cell-mediated immune responses, innate immune responses, pro-inflammatory and pro-fibrotic polarized regulatory T cells, and reduced dendritic cells. Treatment is mainly with glucocorticoids, but the long-term efficacy is not satisfactory. Immunosuppressants, infliximab, rituximab, adalimumab, curcumin, plasma exchange, surgical debulking, or resection may be used in patients unresponsive to systemic glucocorticoids. In addition, the disease has a certain proportion of relapses, and some patients have multiple relapses. It is necessary to give patients confidence, especially for recurrent refractory OIPs. This review aims to deepen our understanding of OIP, further study its pathogenesis, and provide evidence for targeted therapy and reducing the side effects of drug therapy. In particular, the exploration of its specific pathogenesis has not yet reached a unified conclusion, and the development of high-throughput sequencing technology and bioinformatics has provided a means for its exploration. Based on the fact that the pathogenesis of OIP is closely related to T cells, immune repertoire sequencing, such as T cell sequencing, is likely to become the direction of future research. Its application may not only potentially explain T cell clonal dynamics, differentiation, and response trajectories in OIP disease, but also provide unprecedented clues for predicting the effectiveness of treatments with specific drugs such as glucocorticoids or biologics to avoid unnecessary adverse effects.

## Data Availability

Not applicable.

## Abbreviations

OIP: Orbital inflammatory pseudotumor
IOI: Idiopathic orbital inflammation
IOIS: Idiopathic orbital inflammatory syndrome
MRI: Magnetic resonance imaging
CT: Computed tomography
GPA: Granuloma with polyangiitis
PCA: Principal coordinates analysis
GSEA: Gene Set Enrichment Analysis
TLRs: Toll-like receptors
Th1: T helper 1
Tregs: Regulatory T cells
DCs: Dendritic cells
APCs: Antigen-presenting cells
pDCs: Plasmacytoid dendritic cells
cDCs: Conventional dendritic cells
TCR: T cell receptor
EBV: Epstein–Barr virus
EBERs: EBV-encoded small RNAs
miRNAs: MicroRNAs
